# Long-range crossed Andreev reflection in a topological insulator nanowire proximitized by a superconductor

**DOI:** 10.1038/s41567-025-02806-y

**Published:** 2025-03-11

**Authors:** Junya Feng, Henry F. Legg, Mahasweta Bagchi, Daniel Loss, Jelena Klinovaja, Yoichi Ando

**Affiliations:** 1 Physics Institute II, University of Cologne, Cologne, Germany; 2 Department of Physics, University of Basel, Basel, Switzerland; 3 Present Address: SUPA, School of Physics and Astronomy, University of St Andrews, St Andrews, UK

**Keywords:** Topological insulators, Superconducting properties and materials, Superconducting devices

## Abstract

Crossed Andreev reflection is a non-local transport phenomenon that creates and detects Cooper pair correlations between distant locations. It is also the basis of Cooper pair splitting to generate remote entanglement. Although crossed Andreev reflection has been extensively studied in semiconductors proximity-coupled to a superconductor, observing it in a topological insulator has been very difficult. Here we report the observation of this effect in a proximitized topological insulator nanowire. We perform local and non-local conductance spectroscopy on mesoscopic devices in which superconducting niobium and metallic contacts are connected to a bulk-insulating nanowire. In our local conductance measurements we detect a hard gap and the appearance of Andreev bound states that can reach zero bias. We also occasionally observe a negative non-local conductance when sweeping the chemical potential, providing evidence of crossed Andreev reflection. This signal is detected even over length scales much longer than the expected superconducting coherence length of either niobium or the proximitized nanowire. We suggest that this long-range effect is due to the intricate role of disorder in proximitized nanowires.

## Abstract (web summary)

Crossed Andreev reflection is an important process for using the inherent entanglement within a Cooper pair. Now this effect has been observed in a topological insulator nanowire, which has implications for topological quantum computing.

## Main

Topological superconductors^1^ have been a subject of great interest owing to their relevance to non-Abelian Majorana zero modes that are key to topological quantum computation^2^. Proximitized Rashba nanowires (NWs)^3,4^ have been crucial to experimental efforts in this direction^5–7^, but the realization of Majorana zero modes is yet to be confirmed^8–10^, mainly due to disorder and the ambiguity of signatures observed in experimental realizations. Crossed Andreev reflection (CAR), which is a non-local transport phenomenon reflecting superconducting (SC) correlations between two normal-metal leads connected to a superconductor, has been suggested as a probe of bulk topological superconductivity in Rashba NWs due to its expected independence from local physics^7,11–15^, but has not been applied successfully thus far^10^. CAR is also used to create Andreev molecules^16^ and simulate topological superconductivity in the minimal Kitaev chain realized in semiconductor double quantum dots^17^, as well as for Cooper pair splitting^18–21^ in a similar setting.

Topological superconductivity should be more easily realized in topological insulators (TIs)^22^ than in Rashba NWs, because a large Zeeman energy is not necessary to generate a topological gap in TIs. Motivated by this prospect, the SC proximity effect in a TI has been studied in various structures^23–30^. Proximitized TINWs are particularly interesting in this respect, because theory predicts the appearance of Majorana zero modes in the presence of moderate parallel magnetic fields^31–34^. However, experimental studies of the SC proximity effect in TINWs have mostly been performed on Josephson junctions and the information obtained has been limited^28,29,35,36^, with no signatures of CAR. To better understand the SC proximity effect in TINWs, we used a combination of local and non-local conductance spectroscopies^37^ and attempted to detect CAR signatures^38^, as has been done for proximitized Rashba NWs^7,11–15^. In the local conductance, we found that a hard gap can occur, signalling robust proximity-induced superconductivity, along with the appearance of Andreev bound states (ABSs) that arise in the unproximitized normal sections of the NW and can reach zero energy even at zero magnetic field. Our measurements of the non-local conductance showed that our SC-TINW hybrid device works as a reasonably good Cooper pair splitter and that CAR can take place over length scales as long as 1.5 μm. This implies that a SC correlation that is necessary for the CAR process can be established throughout the TINW for a length scale much longer than the expected SC coherence length *ξ*_SC_, which is estimated to be shorter than 110 nm. This long-range CAR effect goes beyond simple descriptions of non-local conductance in mesoscopic superconductors and points to an intricate role of disorder in proximitized NWs; namely, overlapping ABSs created by disorder can lead to an Andreev band that extends beyond *ξ*_SC_ and support long-range CAR^10^. As such, our result is an interesting example counter to the common belief that non-local transport phenomena should be independent of local physics and provide a simple measure of bulk SC properties.

## SC-TINW hybrid device

**Fig. 1:**
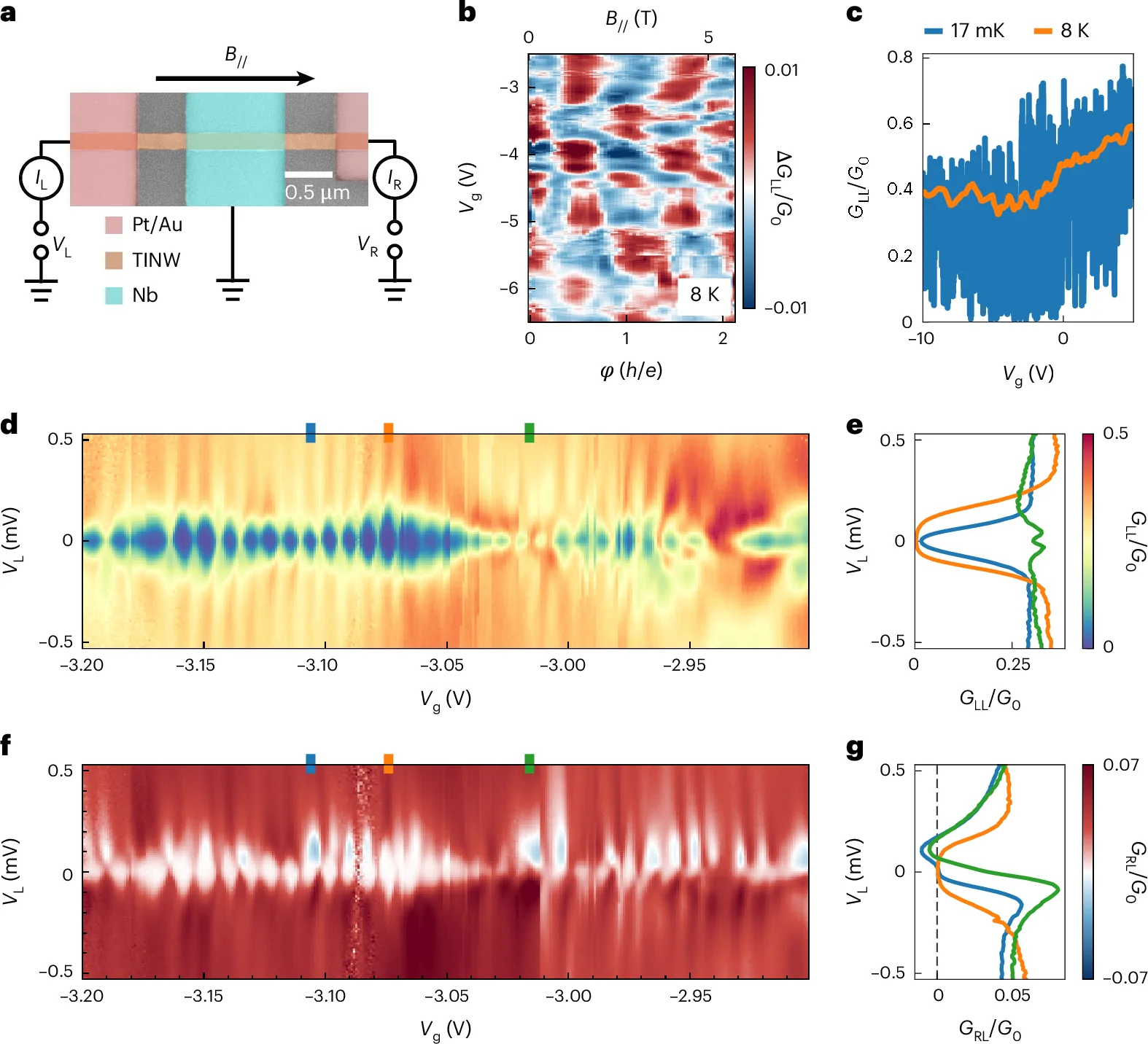
Local and non-local conductance of a TINW proximitized by Nb. **a**, False-colour scanning electron microscope image of the device and schematic of the measurement set-up. Scale bar: 0.5 μm. **b**, Checkerboard pattern in zero-bias *G*_LL_ in the *B*_∥_ versus *V*_g_ plane at 8 K, which is above the SC transition temperature *T*_c_ of the Nb electrode. We plot Δ*G*_LL_, which is calculated by subtracting a smooth *B*_∥_-dependent background (see Supplementary Note 2) from *G*_LL_ to enhance the visibility of the oscillations. The flux generated by *B*_∥_ in the TINW is shown on the bottom *x* axis in units of *h*/*e*, which corresponds to *B*_∥_ = 2.7 T. *G*_0_ ≡ 2*e*^2^/*h*. **c**, *V*_g_ dependence of zero-bias *G*_LL_ at 8 K and 17 mK. In the 8 K data, one can see that *G*_LL_ presents a broad minimum at around *V*_g_ = −5 V, which signals the Dirac point. **d**,**f**, Plots of *G*_LL_ (**d**) and *G*_RL_ (**f**) measured as a function of *V*_L_ and *V*_g_ at 17 mK with *V*_R_ = 0. **e**,**g**, Line cuts of the *G*_LL_ (**e**) and *G*_RL_ (**g**) spectra at three *V*_g_ values indicated by coloured markers (−3.106 V, −3.074 V and −3.016 V) in **d** and **f**. The orange spectrum of *G*_LL_ in **e** is an example of the hard gap. The green and blue spectra of *G*_RL_ in **g** become negative at small positive bias, which is a signature of CAR. Here *V*_L_ is varied and *V*_R_ = 0. Source data

To perform the local and non-local conductance spectroscopy^37^ on a proximitized TINW, we prepared devices as shown in Fig. 1a. We first fabricated a TINW by dry etching^36^ an exfoliated flake (about 15 nm thick) of the bulk-insulating TI material BiSbTeSe_2_ (BSTS)^39^ to obtain a width of 100–200 nm, then deposited SC Nb electrodes and metallic Pt/Au electrodes onto the TINW separated by a gap of about 500 nm. The TINW shown in Fig. 1a was used for most of the measurements reported in this Article and had a cross-section of about 140 × 11 nm^2^. Our finding was essentially reproducible in a total of five similar non-local devices. The local and non-local conductance spectroscopy was performed using the Pt/Au electrodes on either side of a grounded Nb electrode, as depicted in Fig. 1a. The local and non-local differential conductances *G*_*ji*_ ≡ d*I*_*j*_/d*V*_*i*_ (where *i*, *j* = L, R) define a conductance matrix: *V*_*i*_ is the bias voltage applied to the left (L) or right (R) of the TINW via the Pt/Au electrode and *I*_*j*_ is the total current that flows in response to the bias *V*_*i*_. In the experiment, we superimposed a small-amplitude a.c. voltage ${v}_{\mathrm{ac}}^{i}$ of frequency *f*_*i*_ to the d.c. bias *V*_*i*_ to modulate the chemical potential *μ* on the *i* side, and the resulting a.c. component of the current ${i}_{\mathrm{ac}}^{\,j}$ at the *j* electrode was lock-in detected at *f*_*i*_ to calculate the differential conductance ${G}_{ji}\equiv{\mathrm{d}}{I}_{j}/{\mathrm{d}}{V}_{i}={i}_{\mathrm{ac}}^{\,j}/{v}_{\mathrm{ac}}^{i}$. The sign of *I*_*j*_ was defined as positive when the current flows into (out from) the middle electrode for the local (non-local) conductance. The details of the measurement circuit are shown in the Supplementary Note 1. The device had a global back gate. While we assume that *μ* is essentially pinned in the sections covered by metals (Nb and Pt/Au), *μ* in the section between them (bare TINW) is tunable using the back-gate voltage *V*_g_.

At 8 K, where the Nb electrode (*T*_c_ ≈ 6.5 K) is in the normal state, the presence of the subbands characteristic of TINWs can be inferred from the Aharonov–Bohm-like oscillations in *G*_LL_ in parallel magnetic fields *B*_∥_ (refs. ^40,41^). These oscillations occasionally present π-phase shifts as *V*_g_ is changed^42,43^, leading to a checkerboard pattern in the *B*_∥_ versus *V*_g_ plane^36,44^, as shown in Fig. 1b. The periodicity of the Aharonov–Bohm-like oscillations corresponds to the flux quantum *φ*_0_ = *h*/*e* (where *h* is Planck’s constant and *e* is the elementary charge) and the observed periodicity of about 2.7 T is reasonable for the cross-section of our TINW.

## Electron confinement and ABSs

The *V*_g_ dependence of *G*_LL_ at 8 K and at 0 T shown in Fig. 1c indicates that the Dirac point is reached at *V*_g_ ≈ −5 V. At 17 mK, however, we observed that *G*_LL_ measured at zero bias oscillates strongly as a function of *V*_g_ (see Supplementary Note 7 for a zoom-in). To understand these oscillations, we took differential conductance spectra with small *V*_g_ steps (Fig. 1d), which clarified that the strong *G*_LL_ oscillations were due to repeated opening and closing of a gap, which took place with a spacing Δ*V*_g_ of only about 0.01 V. The electron–hole symmetry in *G*_LL_ as a function of *V*_L_ strongly suggests that the origin of the gap is superconductivity; however, the nearly regular gap closing as a function of *V*_g_ is unusual, suggesting that some mesoscopic effect is at work.

**Fig. 2:**
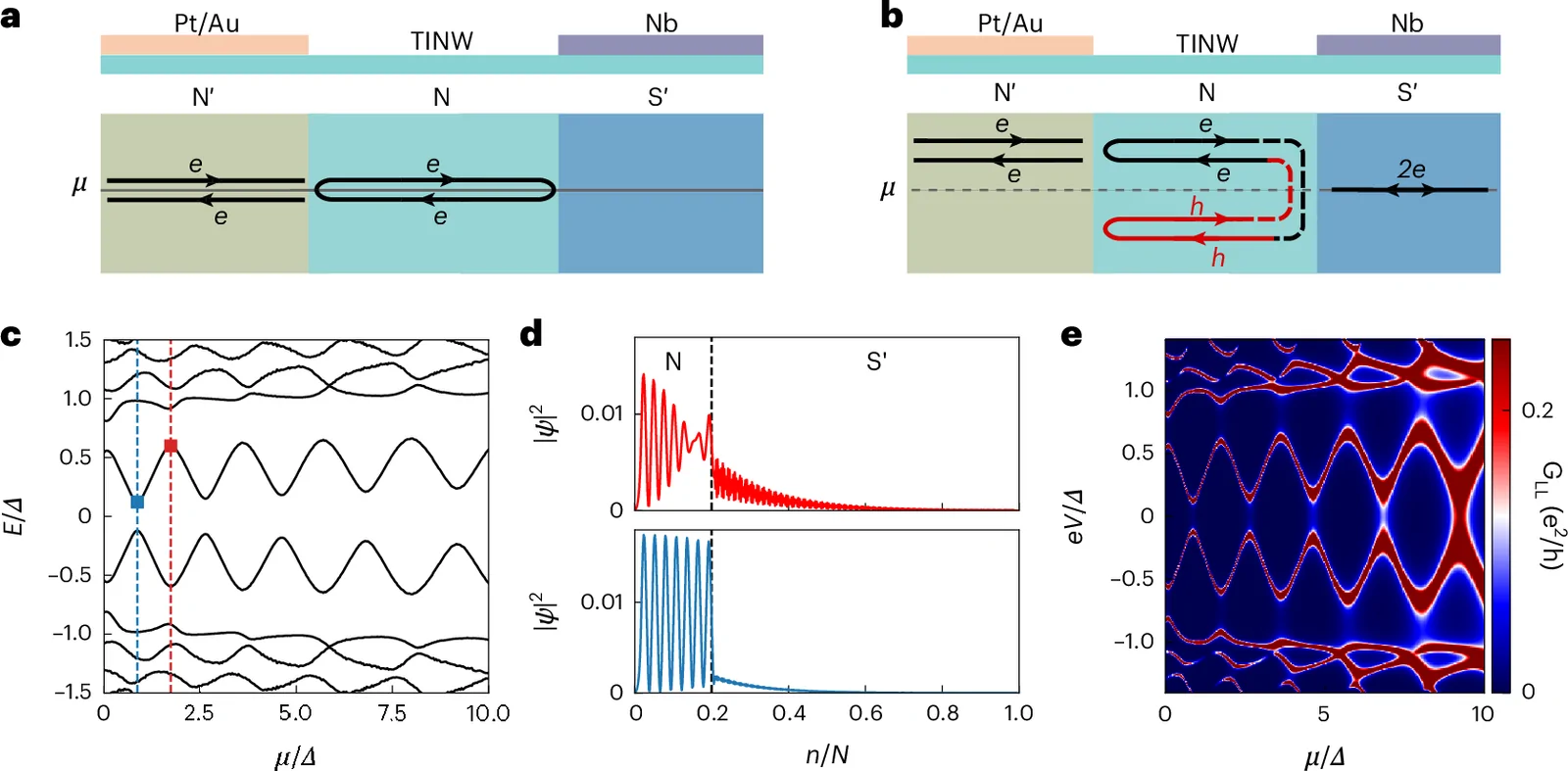
Reflection processes and simulated ABSs in NWs. **a**,**b**, Schematic drawings of the normal reflection and Andreev reflection processes that contribute to the formation and energy of ABSs in the bare section (N) of the TINW. Electron transfer from Pt/Au and from Nb causes the TINW sections beneath them (N′ and S′, respectively) to have different *μ* values, resulting in normal reflection at both interfaces to the N section, as well as Andreev reflection at the NS′ interface. **c**, Simulated energy of an individual ABS in the N region (see Supplementary Note 6 for details); the energy of the low-lying ABS shown here is given by the relative ratio of Andreev reflection and normal reflection. Normal reflection is largest at particular resonant *k*_F_ values that satisfy cos(2*k*_F_*L*) = 0 due to Fabry–Perot-like interference in the quantum well. This results in a minimum of the ABS energy as a function of *μ* at resonance (blue dashed line), and a maximum off resonance (red dashed line). **d**, Top: the wavefunction ψ of the ABS off resonance (red) has a significant weight in the S′ section of the NW (to the right of the black dashed line). Bottom: on resonance, the weight of the ABS in the S′ region is reduced. **e**, Owing to the ABS in the junction region, the simulated *G*_LL_ exhibits oscillations as a function of *μ*. Note that in the experimental set-up there is no explicit tunnel barrier so the broadening of the conductance is primarily dependent on the coupling to the normal lead (here largest for large *μ*). Large broadening of the ABS energy results in a finite conductance at zero-bias. Source data

The strong sensitivity of the gap to *V*_g_ suggests that this gap exists in the section of the TINW not covered by Nb nor Pt/Au; that is, we are probably observing the states in the bare TINW section, which is about 500 nm in length. When the TINW is brought into proximity to Nb, in addition to inducing superconductivity, metallization effects drastically change the properties of the TINW in the region under the Nb contact (for instance, causing a change in *μ* and the Fermi velocity *v*_F_; ref. ^45^). We can also expect similar effects, albeit probably weaker, for the TINW under the Pt/Au contact. The result is that a potential well forms in the bare TINW section between the Pt/Au contacts and Nb (that is, in the N region of the N′NS′ structure between normal lead and SC lead; Fig. 2). Similar to Rashba NWs^46–48^, this potential well results in the formation of ABSs in this bare TINW region. The energy of the ABS depends on the relative strengths of normal reflection and Andreev reflection at the Nb interface (Fig. 2a,b, respectively). Dominant Andreev reflection results in a bound state energy close to the SC gap edge, whereas dominant normal reflection results in a bound state with near zero energy. The origin of the strong *V*_g_ dependence of *G*_LL_ now becomes clear: as *μ* is adjusted, the ratio of normal and Andreev reflections is altered. Indeed, for an ABS resulting from a single subband with Fermi momentum *k*_F_, Fabry–Perot-like interference can result in a large normal reflection when the length *L* of the N section satisfies the resonance condition $2{k}_{{\rm{F}}}L=({N}_{{\rm{FP}}}+\frac{1}{2})\uppi$, when *N*_FP_ is an integer^46,48^. To be more specific, in Fig. 2c we show a simulation of the energy of a single bound state in an NS region (see Supplementary Note 6 for details). As expected, the energy of the ABS oscillates with *μ* within the SC gap depending on the ratio of Andreev and normal reflections. We note that the energy never reaches precisely zero, which at 0 T is possible only for perfect normal reflection^46^. However, in Fig. 2e we show the outcome of an example simulation of *G*_LL_ without a well-defined tunnel barrier to the normal lead: owing to the weak tunnel barrier, a strong coupling of the ABS to the normal lead results in a significant broadening of *G*_LL_ such that it can reach zero bias, as observed in our experiment. Note that the separability of the transverse physics from longitudinal physics in TINW^33^ allows us to use a one-dimensional model for the above simulations; the transverse modes are quantized and indexed by angular momentum, which affects the longitudinal modes only indirectly^33,45^.

One can see that this scenario is consistent with the data: the Dirac point is at *V*_g_ ≈ −5 V (Fig. 1c) and the *V*_g_ separation between resonances, Δ*V*_g_, seen in Fig. 1d near *V*_g_ = −3 V is about 0.01 V. Thus, there should be roughly 200 resonances between −5 V and −3 V. This implies *N*_FP_ ≈ 200 at *V*_g_ = −3 V, giving *k*_F_ ≈ 0.6 nm^−1^ for *L* = 500 nm. With *v*_F_ = 3 × 10^5^ m s^−1^ for the Dirac cone in BSTS^39^, this *k*_F_ corresponds to the Fermi energy *E*_F_ ≈ 0.1 eV, which is in the right ballpark. In addition, the estimate of about 200 resonances for *E*_F_ ≈ 0.1 eV implies an energy interval of about 0.5 meV, which is consistent with our observation that the checkerboard pattern in *G*_LL_(*B*_∥_, *V*_g_) gradually breaks up into more fine-structured patterns for *T* ≲ 4 K (Supplementary Note 3). From the temperature dependence of the Aharonov–Bohm-like conductance oscillations, we estimated a phase-coherence length *L*_p_ of 1.9 μm in our TINW (Supplementary Note 3). This supports the existence of Fabry–Perot-like interference in the 500-nm-long bare TINW.

## Non-local conductance and CAR

**Fig. 3:**
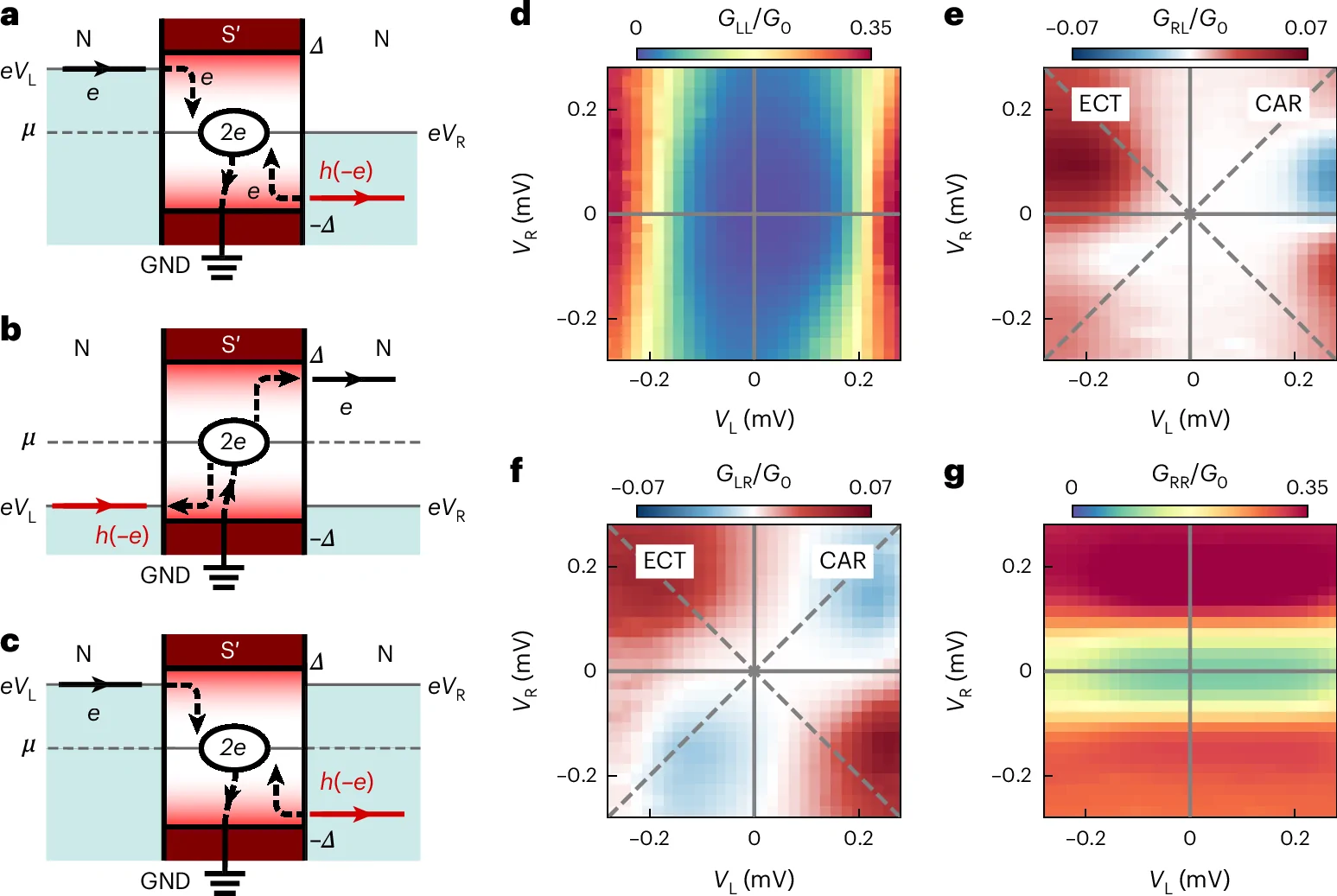
Non-local processes involving Cooper pairs and conductance matrix as a function of *V*_L_ and *V*_R_. **a**, Schematic drawing of the CAR process in S′ contacted by N on both sides. Red shading indicates the presence of Andreev/bulk states within S′, which itself is proximitized by Nb. An electron with energy *eV*_L_ coming from the left N creates a Cooper pair in S′ by taking an electron from the right N, emitting a hole with energy −*eV*_L_ to the right. The created Cooper pair is drained to the ground (GND). **b**, Cooper pair splitting: when S′ in the middle is positively biased, the probability of splitting a Cooper pair (provided from the ground) into the two N contacts is maximal. **c**, Cooper pair creation: when S′ is negatively biased, the probability of creating a Cooper pair in S′ by taking electrons from the two N contacts is maximal, and the Cooper pair is drained to the ground. The Cooper pair splitting and creation are microscopically the CAR process. **d**–**g**, *G*_LL_ (**d**), *G*_RL_ (**e**), *G*_LR_ (**f**) and *G*_RR_ (**g**) measured at 17 mK by varying both *V*_L_ and *V*_R_ at fixed *V*_g_ = −4.368 V. Note the different colour scales for local and non-local conductances. The dashed grey lines in **e** and **f** mark *V*_L_ = *V*_R_ and *V*_L_ = −*V*_R_. CAR is enhanced when *V*_L_ and *V*_R_ have the same sign, whereas opposite-sign *V*_L_ and *V*_R_ promote ECT^49^. The behaviour of *G*_LR_ in **f** indeed follows this expectation. The *G*_RL_ data in **e** slightly deviate from this, which is probably due to the non-negligible *G*_RR_ at *V*_R_ = 0 that would enhance ECT. The data for *G*_LL_ and *G*_RR_ in **d** and **g** confirm that the local conductances are not affected by the bias on the other side. Source data

While the local *G*_LL_ seems to be mainly governed by the states in the N section of our device, the non-local *G*_RL_ contains information on the S′ section. The spectra of *G*_RL_(*V*_L_) measured for the same *V*_g_ range are plotted in Fig. 1f (the bias was applied only to the left lead; namely, *V*_R_ = 0 for this measurement). One can see that at zero bias, *G*_RL_ vanishes when *V*_g_ is off-resonance, while a positive *G*_RL_ is observed on-resonance. This positive *G*_RL_ is most probably due to the process called elastic co-tunnelling (ECT), in which an electron tunnels between the N sections on the left and right of S′; as an electron injected from the left comes out as an electron on the right, *G*_RL_ is positive in the case of ECT. CAR is a competing process in which an electron injected into S′ from the left forms a Cooper pair by taking an electron from the right of S′, ejecting a hole into the right N section; this hole causes a negative *G*_RL_ and the Cooper pair created in S′ is drained to the ground (Fig. 3a). The CAR process requires a state that is extended throughout the S′ part^11^, and therefore CAR provides unambiguous evidence for the SC correlation in S′. Our data in Fig. 1f show that CAR can become dominant at the *V*_g_ values indicated by blue and green markers, where *G*_LL_ (Fig. 1e) suggests the existence of a sufficient number of states at *V*_L_ > 0 on the left of S′. The negative *G*_RL_ in Fig. 1g is a clear signature of CAR in a proximitized TINW. Note that, as a non-local process, the occurrence of CAR also depends on the ABSs on the right of S′, which is reflected in *G*_RR_. The orange marker labels an off-resonance *V*_g_ position (Fig. 1d), where the absence of states at low bias (Fig. 1e) leads to *G*_RL_ ≈ 0 (Fig. 1g). The length of S′ is 900 nm here, while the SC coherence length ${\xi}_{{\rm{SC}}^{\rm{Nb}}}$ in our Nb is about 30 nm (ref. ^30^), meaning that CAR must be taking place through the proximitized TINW, not through Nb.

**Fig. 4:**
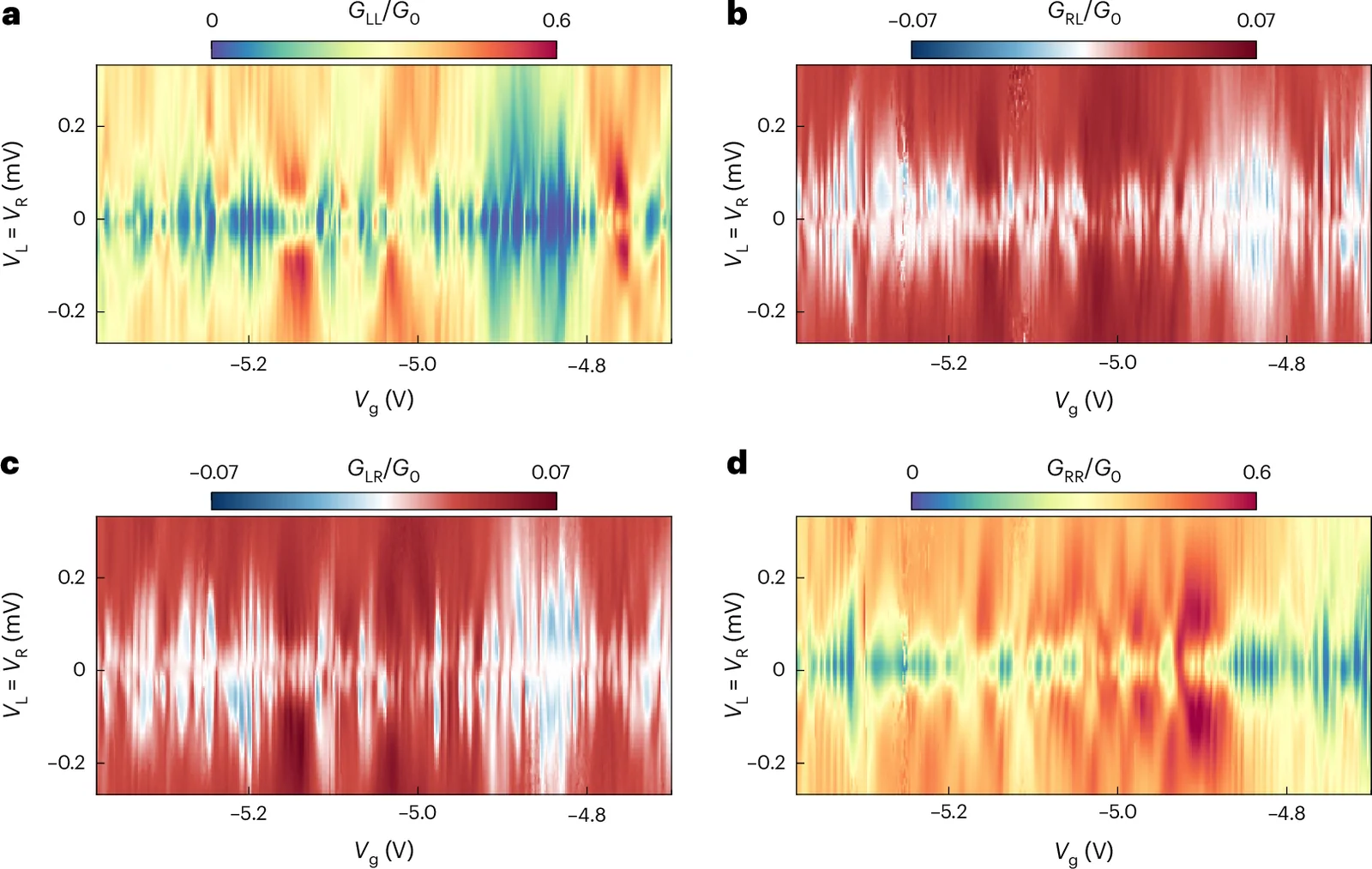
Conductance matrix measured with *V*_R_ = *V*_L_ at zero magnetic field. **a**–**d**, *G*_LL_ (**a**), *G*_RL_ (**b**), *G*_LR_ (**c**) and *G*_RR_ (**d**) measured at 17 mK and 0 T as a function of *V*_g_ and *V*_L_ (=*V*_R_). The *V*_g_ range shown here is near the Dirac point. For the measurement of *G*_RL_, *V*_L_ is modulated with a frequency *f*_L_ and the a.c. component in *I*_R_ at the frequency *f*_L_ is lock-in detected. In this way, *G*_RL_ measured on the right reflects the a.c. drive on the left. The same is true for *G*_LR_. Source data

The competition between ECT and CAR can be manipulated by applying a finite bias not only to the left, but also to the right (that is making *V*_R_ ≠ 0)^49^. For example, when *V*_L_ = *V*_R_ < 0, the process of splitting a Cooper pair in S′ into one electron on each side (Fig. 3b) is enhanced. This Cooper pair splitting is microscopically the same process as CAR and it gives rise to negative *G*_RL_ and *G*_LR_. The inverse of the Cooper pair splitting (that is, electrons from either side creating a Cooper pair in S′; Fig. 3c) is promoted when *V*_L_ = *V*_R_ > 0, and this process also yields negative *G*_RL_ and *G*_LR_. On the other hand, with *V*_L_ = −*V*_R_, the process by which an electron on the high-bias side tunnels through S′ to the low-bias side is enhanced; this is the ECT process microscopically and yields positive *G*_RL_ and *G*_LR_. In our device, we measured all four differential conductances (*G*_LL_, *G*_RL_, *G*_LR_ and *G*_RR_) in the *V*_L_ versus *V*_R_ plane at a fixed global *V*_g_. The results shown in Fig. 3d–g, obtained with a *V*_g_ where *G*_LL_ is off-resonance and *G*_RR_ is relatively small, confirm the expected controllability of the competition, although the behaviour in Fig. 3e is less ideal (Supplementary Note 8). Taking advantage of this controllability, we measured the full conductance matrix by setting *V*_L_ = *V*_R_ to enhance CAR and the results are shown in Fig. 4, which presents a *V*_g_ range near the Dirac point. One can see that negative *G*_LR_ and *G*_RL_ signalling the CAR process appear frequently upon sweeping *V*_g_. The range of *V*_g_ where CAR occurs is broadly consistent between *G*_RL_ and *G*_LR_, confirming that this process reflects the SC coherence throughout the S′ section of the TINW.

## Effects of parallel magnetic fields

**Fig. 5:**
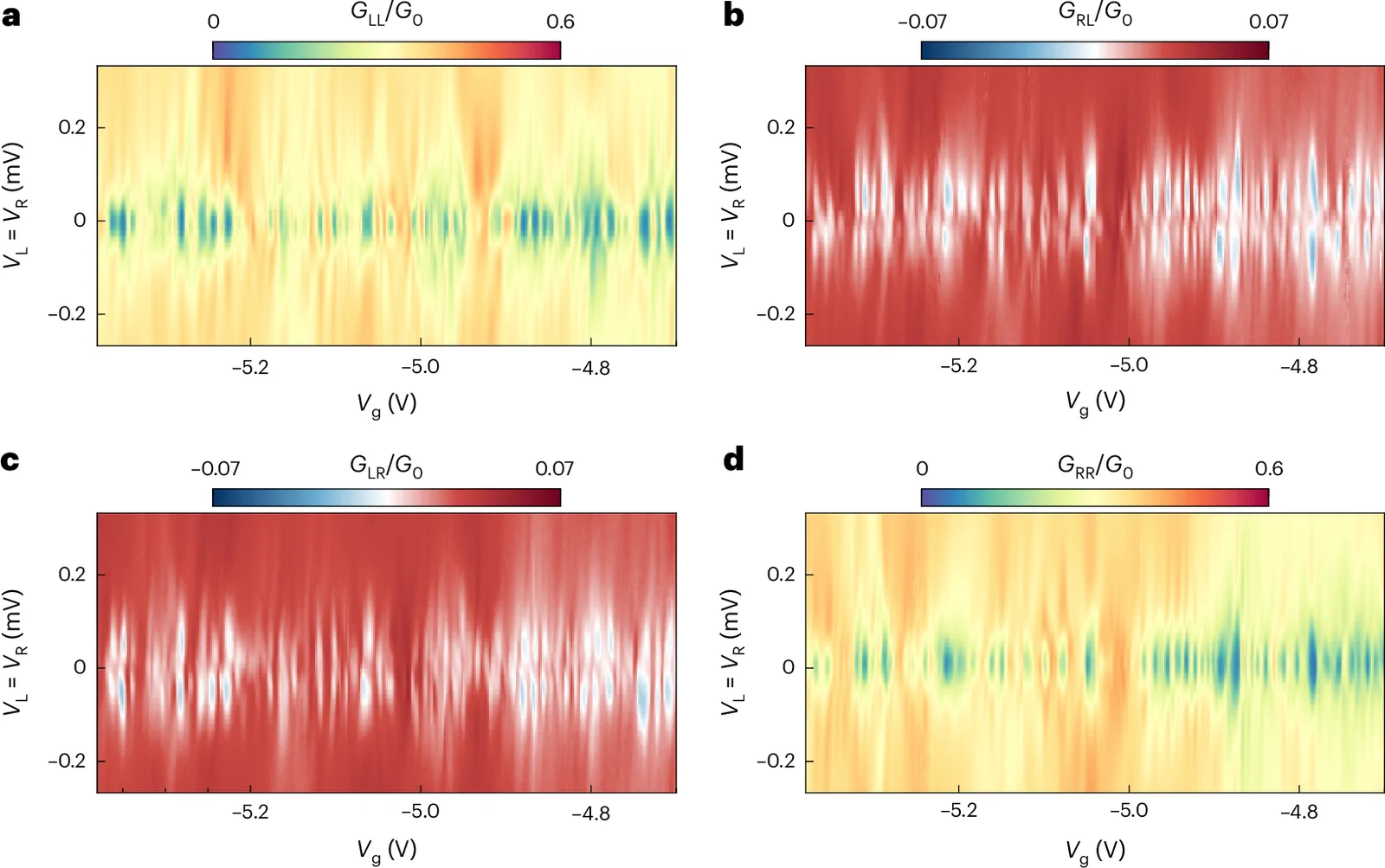
Conductance matrix measured with *V*_R_ = *V*_L_ for $\varphi =\frac{1}{2}(h/e)$. **a**–**d**, *G*_LL_ (**a**), *G*_RL_ (**b**), *G*_LR_ (**c**) and *G*_RR_ (**d**) measured in a parallel magnetic field of 1.35 T, which generates the magnetic flux $\varphi =\frac{1}{2}(h/e)$ in the TINW. The gaps in *G*_LL_ and *G*_RR_ due to the ABS, as well as the CAR signal in *G*_LR_ and *G*_RL_, are both observed at this flux, implying that the proximity-induced superconductivity is not destroyed by the angular-momentum mismatch. Nevertheless, the spectral features are weakened because of the softer SC gap. Source data

A parallel magnetic field generates a magnetic flux *φ* in the TINW and modulates the electronic structure in a non-trivial way. In particular, the subband gap closes and a spin-non-degenerate band appears at $\varphi =(n+\frac{1}{2})h/e$. This change is expected to have the following implications for the proximity effect: on the one hand, it has been suggested that the odd number of bands is ideal for inducing topological superconductivity^31^; on the other hand, the flux of $(n+\frac{1}{2})h/e$ shifts the angular momentum of the subbands in such a way that the Cooper pairing becomes forbidden (called angular-momentum mismatch)^32^, at least in a system with rotational symmetry. To observe the effects of $\varphi =\frac{1}{2}(h/e)$ in our device, we measured the full conductance matrix at this flux with *V*_L_ = *V*_R_ for the same *V*_g_ range (Fig. 5). One can see that there is no drastic change in the data and the key features—the periodic gap opening and closing and the existence of CAR—are still observed at $\varphi =\frac{1}{2}(h/e)$. Looking more closely, the gap becomes soft and CAR is observed less frequently, which is consistent with a reduced (but non-zero) SC gap. The *φ* dependence of *G*_*ij*_ for a range of *V*_g_ at a fixed bias is also consistent with a reduced gap near $\varphi =\frac{1}{2}(h/e)$ (Supplementary Note 4).

Our data for $\varphi =\frac{1}{2}(h/e)$ clearly show that the SC gap does not close at this flux, which implies that the angular-momentum mismatch does not completely prohibit the SC gap in our TINW. This result confirms the prediction^32–34,45^ that the inversion symmetry breaking caused by having the SC contact on only the top surface or gating from one side is sufficient to mitigate the mismatch problem. When Cooper pairing is present in the TINW at $\varphi =\frac{1}{2}(h/e)$, one would expect the appearance of a topological SC state accompanied by Majorana bound states (MBSs) at any chemical potential^31^. However, we did not observe a stable zero-bias conductance peak that would signal the MBSs. This absence of a robust zero-bias conductance peak in our experiment does not necessarily mean that a topological SC state is not induced in our TINWs. This is because the presence of the long N sections next to the SC section, as well as the possible hybridization of the MBSs through the Andreev band, would obscure the peak due to MBSs. As there is considerable room for improvement in hybrid TINW devices that are more tolerant of disorder than Rashba NW devices^33^, it would be interesting to pursue the realization of MBSs in TINWs.

## Devices with different S′ section lengths

**Fig. 6:**
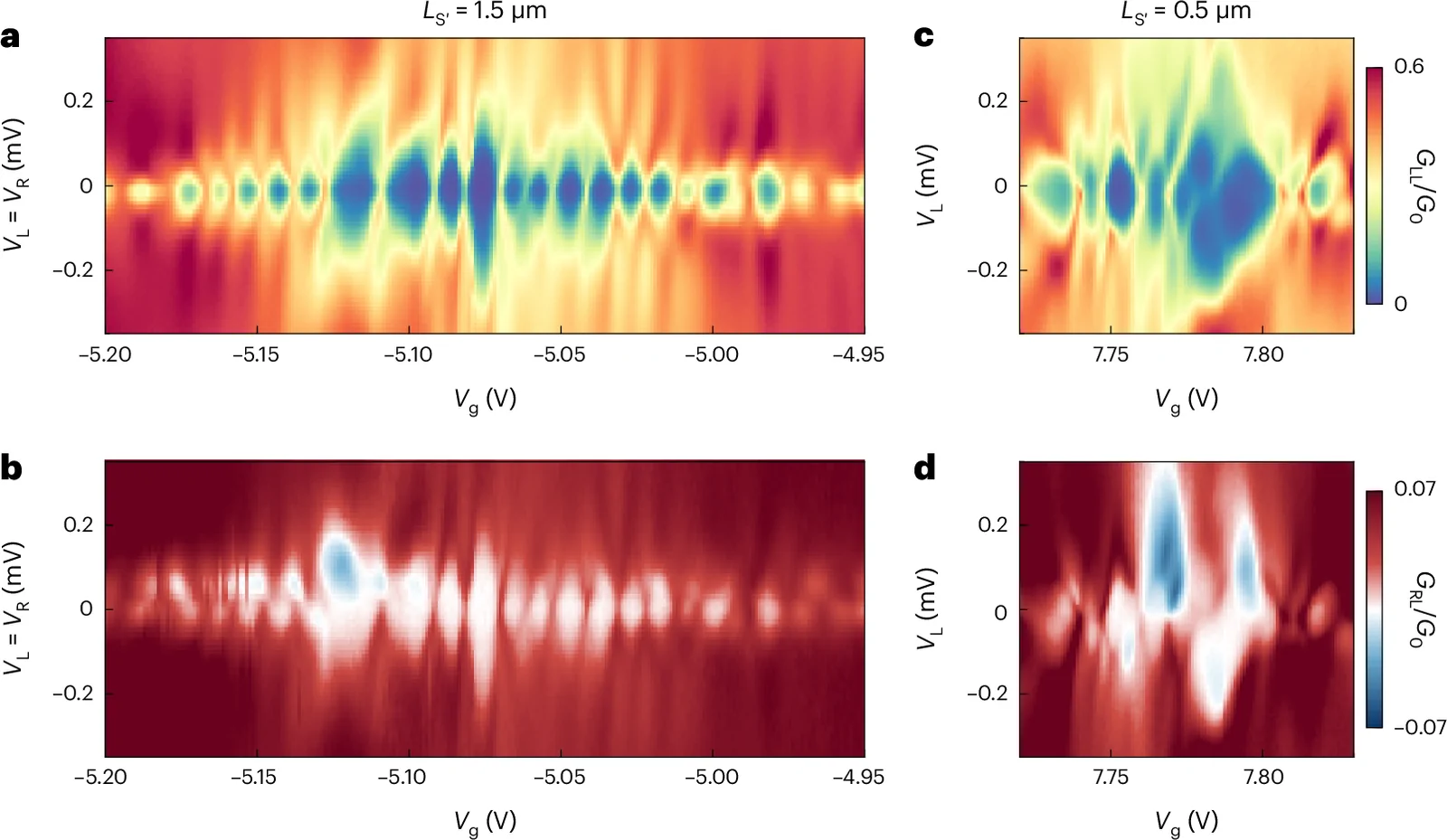
*G*_LL_ and *G*_RL_ in devices with different *L*_S′_. **a**,**b**, *G*_LL_ (**a**) and *G*_RL_ (**b**) in a device with *L*_S′_ = 1.5 μm measured with *V*_R_ = *V*_L_ for a *V*_g_ range from −5.20 to −4.95 V. **c**,**d**, *G*_LL_ (**c**) and *G*_RL_ (**d**) in another device with *L*_S′_ = 0.5 μm for a *V*_g_ range from 7.72 to 7.83 V measured by applying *V*_L_ only (that is, *V*_R_ = 0). The sizes of the horizontal axes are tuned such that the same horizontal length in the plots corresponds to the same *V*_g_ range for the two datasets. Note that the *V*_g_ distance between the resonances is essentially independent of *L*_S′_. Negative *G*_RL_ was observed even for *L*_S′_ = 1.5 μm, whereas *ξ*_SC_ is estimated to be shorter than 110 nm. The colour scale in **c** (**d**) also applies to **a** (**b**). Source data

To understand how the local and non-local conductances change when the length of the S′ section (*L*_S__′_) varies, in Fig. 6 we present the results for *G*_LL_ and *G*_RL_ for devices with *L*_S__′_ = 1.5 and 0.5 μm. First, the *V*_g_ distance between the resonances in both devices is nearly the same as that in the *L*_S′_ = 900 nm device. This supports our interpretation that the gap closing is due to the Fabry–Perot-like resonance in the N section, the length of which was always around 500 nm. Second, the CAR signal was observed in the *G*_RL_ data of even the device with *L*_S__′_ = 1.5 μm, which means that the SC correlation in the S′ section extends to at least 1.5 μm (see Supplementary Note 5 for additional data).

To examine the implications of this observation, we estimated *ξ*_SC_ in the TINW. If we took the observed gap of about 0.2 meV seen in the orange curve in Fig. 1e as the lower bound of *Δ* (which can be larger if the orange curve is dominated by in-gap states in N or S′), the upper bound of the clean-limit BCS coherence length *ξ*_BCS_ = *ℏv*_F_/(π*Δ*) is estimated to be about 0.3 μm, which is already shorter than 1.5 μm. Given that our BSTS has a mean free path *ℓ*_mfp_ ≈ 40 nm (ref. ^50^), a cautious estimate gave ${\xi }_{{\rm{SC}}}=\sqrt{{\xi }_{{\rm{BCS}}}{\ell }_{{\rm{mfp}}}}$ of at most 110 nm. Therefore, our 1.5-μm device shows that CAR can be observed for length scales more than ten times longer than *ξ*_SC_. At first sight, such a long-range CAR effect is surprising, as it suggests the existence of subgap states that have a spatial extent much longer than *ξ*_SC_. In this regard, it was recently shown theoretically that quasiperiodic disorder in a proximitized NW can create overlapping ABSs that form a band extending throughout the NW and result in a long-range non-local conductance that can have a dominant CAR contribution^10^. Our observation points to the existence of such overlapping ABSs in the S′ region, connecting the left and right N regions coherently for the CAR to take place. For ${L}_{{{\rm{S}}}^{{\prime} }}\simeq 10\,{\xi }_{{\rm{SC}}}$ observed in our TINWs, this would require only a few ABSs to overlap. This scenario seems highly plausible in our set-up, as the relatively high level of disorder^51^ in our compensated TINWs probably results in a considerable number of local ABSs when proximitized.

## Discussion

The Coulomb blockade in a quantum dot can open a hard gap, and hence one might think that it would be premature to conclude that the hard gap we observed is due to superconductivity. In this regard, the *G*_LL_ spectra shown in Fig. 1d show that the on-resonance states appear vertically in this plot and do not disperse with *V*_g_, particularly in the region where the resonances appear regularly. This behaviour is inconsistent with the Coulomb blockade and supports the Fabry–Perot-like origin of the gap closing.

The observation of the CAR process in a TI platform was reported recently in a ferromagnetic TI realizing the quantum anomalous Hall effect, where the CAR occurred across a narrow Nb electrode contacting the chiral edge state^30^. Combined with our result, it seems that a dominant CAR contribution is more easily achieved when the transport channel is one-dimensional.

It is worth mentioning that the non-local conductance is sometimes considered to be free from complications from local physics and allows the topological phase throughout the NW to be probed^7^. In this regard, our data show that *G*_RL_ and *G*_LR_ are primarily governed by the local states formed in the N sections, and that non-local physics plays a secondary role in determining them. Nevertheless, it is interesting to see that when the local states in the N sections allow the CAR process to take place, our device can work as a Cooper pair splitter. If we define the Cooper-pair-splitting efficiency ${\eta }_{{\rm{CPS}}}\equiv {i}_{ac}^{j}/{i}_{ac}^{i}$ (*i* ≠ *j*) for the ${v}_{ac}^{i}$ drive with frequency *f*_*i*_, we obtain *η*_CPS_ of up to 0.5 for our *L*_S′_ = 900 nm device. This is not as high as that obtained in dedicated Cooper pair splitters (up to 0.9)^52^, but is comparable to the efficiencies of other devices^17,49^.

Overall, our data demonstrate that non-local transport in mesoscopic SC systems reflects a complex interplay of local and global properties. Understanding this interplay in the presence of disorder is vital to use non-local transport as a probe of exotic proximity-induced superconductivity—not only in TINWs, but also in other hybrid platforms.

## Methods

### Device fabrication

Large BSTS flakes were exfoliated from a bulk single crystal and then selected pieces were transferred using a polymer to a doped Si substrate with a 290-nm-thick SiO_2_ coating layer. We used electron-beam lithography to define the NW pattern and Ar dry etching to fabricate NWs from the flakes. Nb leads and Pt/Au leads were fabricated separately with a standard electron-beam lithography process and ultra-high-vacuum sputter deposition; a small overlay error between the two steps caused slight differences in the lengths of the N regions. Before metallization, the NW surface was cleaned by ex situ O_2_ etching and in situ Ar-plasma cleaning. Putting the N regions of TINW between Nb and Pt/Au enabled the tunability of *G*_LL_ and *G*_RR_, which is important for the observation of CAR (it is difficult to observe CAR with non-tunable tunnel barriers).

### Measurements

The devices were measured in an Oxford Instruments Triton 300 dilution refrigerator equipped with a 6-1-1-T SC vector magnet. A standard low-frequency lock-in technique with d.c. bias was used to measure the differential conductance (see Supplementary Note 1 for details). For non-local measurements, we used frequencies *f*_L_ and *f*_R_ for the left and right bias modulations, respectively, and measured ${i}_{\mathrm{ac}}^{\mathrm{R}}$ at *f*_L_, such that ${G}_{{\rm{RL}}}={i}_{\mathrm{ac}}^{\mathrm{R}}/{v}_{\mathrm{ac}}^{\mathrm{L}}$ is made to reflect the bias drive on the left; the same is true for *G*_LR_.

### Theory

Simulations were performed using the Python package KWANT^53^ for a one-dimensional NW with a single subband and different *μ* between S′ or N′ and N regions (see Supplementary Note 6 for details). Note that the surface-state wavefunction of the TINW decays almost entirely within about 3 nm (ref. ^45^), forming the shape of a rectangular tube; the analysis in ref. ^45^ suggested that its thickness is not strongly modified by the contact with Nb or gating.

## Online content

Any methods, additional references, Nature Portfolio reporting summaries, source data, extended data, supplementary information, acknowledgements, peer review information; details of author contributions and competing interests; and statements of data and code availability are available at 10.1038/s41567-025-02806-y.

## Supplementary information

Supplementary Information Supplementary Figs. 1–7 and Notes 1–8.

Supplementary Data 1 Source data for Supplementary Figs. 2–5 and 7.

## Source data

Source Data Fig. 1 The file ‘SEM_SC.tif’ is the unprocessed SEM image used in Fig. 1a. Among the other eight text files, the files named ‘raw’ contain the raw data and the files named ‘plot’ contain the processed data to make the plots: Figure_1b_plot_dim=2_.txt, Figure_1b_raw_dim=2_.txt, Figure_1c_1_plot_dim=1_.txt, Figure_1c_1_raw_dim=1_.txt, Figure_1c_2_plot_dim=1_.txt, Figure_1c_2_raw_dim=1_.txt, Figure_1d_f_plot_dim=2_.txt, Figure_1d_f_raw_dim=2_.txt.

Source Data Fig. 2 The following three files contain the data to plot Fig. 2: Figure_2c_dim=2_.txt, Figure_2d_dim=1_.txt, Figure_2e_dim=2_.txt.

Source Data Fig. 3 Among the two text files, the file named ‘raw’ contains the raw data and the file named ‘plot’ contains the processed data to make the plots: Figure_3_plot_dim=2_.txt, Figure_3_raw_dim=2_.txt.

Source Data Fig. 4 Among the two text files, the file named ‘raw’ contains the raw data and the file named ‘plot’ contains the processed data to make the plots: Figure_4_plot_dim=2_.txt, Figure_4_raw_dim=2_.txt.

Source Data Fig. 5 Among the two text files, the file named ‘raw’ contains the raw data and the file named ‘plot’ contains the processed data to make the plots: Figure_5_plot_dim=2_.txt, Figure_5_raw_dim=2_.txt.

Source Data Fig. 6 Among the following four text files, the files named ‘raw’ contain the raw data and the files named ‘plot’ contain the processed data to make the plots: Figure_6a_b_plot_dim=2_.txt, Figure_6a_b_raw_dim=2_.txt, Figure_6c_d_plot_dim=2_.txt, Figure_6c_d_raw_dim=2_.txt.

## Data Availability

Source data are provided with this paper. Raw data used to generate Figs. 1–6 and Supplementary Figs. 2–5 and 7 are available via Zenodo at 10.5281/zenodo.12611921 (ref. ^54^).

## Code availability

The codes used in the theoretical simulations are available via Zenodo at 10.5281/zenodo.12611921 (ref. ^54^).

## References

[1] Sato, M. & Ando, Y. Topological superconductors: a review. *Rep. Prog. Phys.* **80**, 076501 (2017). 28367833 10.1088/1361-6633/aa6ac7

[2] Nayak, C., Stern, A., Freedman, M. & Das Sarma, S. Non-Abelian anyons and topological quantum computation. *Rev. Mod. Phys.* **80**, 1083–1159 (2008).

[3] Oreg, Y., Refael, G. & von Oppen, F. Helical liquids and Majorana bound states in quantum wires. *Phys. Rev. Lett.* **105**, 177002 (2010). 21231073 10.1103/PhysRevLett.105.177002

[4] Lutchyn, R. M., Sau, J. D. & Das Sarma, S. Majorana fermions and a topological phase transition in semiconductor-superconductor heterostructures. *Phys. Rev. Lett.* **105**, 077001 (2010). 20868069 10.1103/PhysRevLett.105.077001

[5] Prada, E. et al. From Andreev to Majorana bound states in hybrid superconductor–semiconductor nanowires. *Nat. Rev. Phys.* **2**, 575–594 (2020).

[6] Flensberg, K., von Oppen, F. & Stern, A. Engineered platforms for topological superconductivity and Majorana zero modes. *Nat. Rev. Mater.* **6**, 944–958 (2021).

[7] Aghaee, M. et al. InAs-Al hybrid devices passing the topological gap protocol. *Phys. Rev. B* **107**, 245423 (2023).

[8] Valentini, M. et al. Nontopological zero-bias peaks in full-shell nanowires induced by flux-tunable Andreev states. *Science* **373**, 82–88 (2021). 34210881 10.1126/science.abf1513

[9] Das Sarma, S. In search of Majorana. *Nat. Phys.* **19**, 165–170 (2023).

[10] Hess, R., Legg, H. F., Loss, D. & Klinovaja, J. Trivial Andreev band mimicking topological bulk gap reopening in the nonlocal conductance of long Rashba nanowires. *Phys. Rev. Lett.* **130**, 207001 (2023). 37267549 10.1103/PhysRevLett.130.207001

[11] Rosdahl, T. O., Vuik, A., Kjaergaard, M. & Akhmerov, A. R. Andreev rectifier: a nonlocal conductance signature of topological phase transitions. *Phys. Rev. B* **97**, 045421 (2018).

[12] Puglia, D. et al. Closing of the induced gap in a hybrid superconductor-semiconductor nanowire. *Phys. Rev. B* **103**, 235201 (2021).

[13] Pan, H., Sau, J. D. & Das Sarma, S. Three-terminal nonlocal conductance in Majorana nanowires: distinguishing topological and trivial in realistic systems with disorder and inhomogeneous potential. *Phys. Rev. B* **103**, 014513 (2021).

[14] Hess, R., Legg, H. F., Loss, D. & Klinovaja, J. Local and nonlocal quantum transport due to Andreev bound states in finite Rashba nanowires with superconducting and normal sections. *Phys. Rev. B* **104**, 075405 (2021).

[15] Banerjee, A. et al. Local and nonlocal transport spectroscopy in planar Josephson junctions. *Phys. Rev. Lett.* **130**, 096202 (2023). 36930915 10.1103/PhysRevLett.130.096202

[16] Su, Z. et al. Andreev molecules in semiconductor nanowire double quantum dots. *Nat. Commun.* **8**, 585 (2017). 28928420 10.1038/s41467-017-00665-7 PMC5605684

[17] Dvir, T. et al. Realization of a minimal Kitaev chain in coupled quantum dots. *Nature* **614**, 445–450 (2023). 36792741 10.1038/s41586-022-05585-1

[18] Recher, P., Sukhorukov, E. V. & Loss, D. Andreev tunneling, Coulomb blockade, and resonant transport of nonlocal spin-entangled electrons. *Phys. Rev. B* **63**, 165314 (2001).

[19] Hofstetter, L., Csonka, S., Nygård, J. & Schönenberger, C. Cooper pair splitter realized in a two-quantum-dot Y-junction. *Nature* **461**, 960–963 (2009). 19829377 10.1038/nature08432

[20] Sato, K., Loss, D. & Tserkovnyak, Y. Cooper-pair injection into quantum spin Hall insulators. *Phys. Rev. Lett.* **105**, 226401 (2010). 21231401 10.1103/PhysRevLett.105.226401

[21] Deacon, R. S. et al. Cooper pair splitting in parallel quantum dot Josephson junctions. *Nat. Commun.* **6**, 7446 (2015). 26130172 10.1038/ncomms8446 PMC4506998

[22] Fu, L. & Kane, C. L. Superconducting proximity effect and Majorana fermions at the surface of a topological insulator. *Phys. Rev. Lett.* **100**, 096407 (2008). 18352737 10.1103/PhysRevLett.100.096407

[23] Zhang, D. et al. Superconducting proximity effect and possible evidence for Pearl vortices in a candidate topological insulator. *Phys. Rev. B* **84**, 165120 (2011).

[24] Maier, L. et al. Induced superconductivity in the three-dimensional topological insulator HgTe. *Phys. Rev. Lett.* **109**, 186806 (2012). 23215314 10.1103/PhysRevLett.109.186806

[25] Williams, J. R. et al. Unconventional Josephson effect in hybrid superconductor-topological insulator devices. *Phys. Rev. Lett.* **109**, 056803 (2012). 23006196 10.1103/PhysRevLett.109.056803

[26] Finck, A., Kurter, C., Hor, Y. & Van Harlingen, D. Phase coherence and Andreev reflection in topological insulator devices. *Phys. Rev. X* **4**, 041022 (2014).

[27] Hart, S. et al. Induced superconductivity in the quantum spin Hall edge. *Nat. Phys.* **10**, 638–643 (2014).

[28] Rosenbach, D. et al. Reappearance of first Shapiro step in narrow topological Josephson junctions. *Sci. Adv.* **7**, eabf1854 (2021). 34162537 10.1126/sciadv.abf1854 PMC8221618

[29] Bai, M. et al. Proximity-induced superconductivity in (Bi_1−x_Sb_x_)_2_Te_3_ topological-insulator nanowires. *Commun. Mater.* **3**, 20 (2022).

[30] Uday, A. et al. Induced superconducting correlations in the quantum anomalous Hall insulator. *Nat. Phys.* **20**, 1589–1595 (2024). 39416854 10.1038/s41567-024-02574-1 PMC11473362

[31] Cook, A. & Franz, M. Majorana fermions in a topological-insulator nanowire proximity-coupled to an *s*-wave superconductor. *Phys. Rev. B* **84**, 201105 (2011).

[32] de Juan, F., Bardarson, J. H. & Ilan, R. Conditions for fully gapped topological superconductivity in topological insulator nanowires. *SciPost Phys.* **6**, 060 (2019). 10.1103/PhysRevLett.113.107003 25238379

[33] Legg, H. F., Loss, D. & Klinovaja, J. Majorana bound states in topological insulators without a vortex. *Phys. Rev. B* **104**, 165405 (2021).

[34] Heffels, D. et al. Robust and fragile Majorana bound states in proximitized topological insulator nanoribbons. *Nanomaterials* **13**, 723 (2023). 36839091 10.3390/nano13040723 PMC9967168

[35] Fischer, R. et al. 4*π*-periodic supercurrent tuned by an axial magnetic flux in topological insulator nanowires. *Phys. Rev. Res.* **4**, 013087 (2022).

[36] Rößler, M. et al. Top-down fabrication of bulk-insulating topological insulator nanowires for quantum devices. *Nano Lett.* **23**, 2846–2853 (2023). 36976857 10.1021/acs.nanolett.3c00169

[37] Gramich, J., Baumgartner, A. & Schönenberger, C. Andreev bound states probed in three-terminal quantum dots. *Phys. Rev. B* **96**, 195418 (2017).

[38] Fuchs, J., Barth, M., Gorini, C., Adagideli, I. & Richter, K. Crossed Andreev reflection in topological insulator nanowire T junctions. *Phys. Rev. B* **104**, 085415 (2021).

[39] Arakane, T. et al. Tunable Dirac cone in the topological insulator Bi_2−*x*_Sb_*x*_Te_3−*y*_Se_*y*_. *Nat. Commun.* **3**, 636 (2012). 22273674 10.1038/ncomms1639

[40] Peng, H. et al. Aharonov–Bohm interference in topological insulator nanoribbons. *Nat. Mater.* **9**, 225–229 (2010). 20010826 10.1038/nmat2609

[41] Zhang, Y. & Vishwanath, A. Anomalous Aharonov-Bohm conductance oscillations from topological insulator surface states. *Phys. Rev. Lett.* **105**, 206601 (2010). 21231253 10.1103/PhysRevLett.105.206601

[42] Cho, S. et al. Aharonov–Bohm oscillations in a quasi-ballistic three-dimensional topological insulator nanowire. *Nat. Commun.* **6**, 7634 (2015). 26158768 10.1038/ncomms8634

[43] Jauregui, L. A., Pettes, M. T., Rokhinson, L. P., Shi, L. & Chen, Y. P. Magnetic field-induced helical mode and topological transitions in a topological insulator nanoribbon. *Nat. Nanotechnol.* **11**, 345–351 (2016). 26780658 10.1038/nnano.2015.293

[44] Kim, H.-S. et al. Adjustable quantum interference oscillations in Sb-doped Bi_2_Se_3_ topological insulator nanoribbons. *ACS Nano* **14**, 14118–14125 (2020). 33030335 10.1021/acsnano.0c06892

[45] Legg, H. F., Loss, D. & Klinovaja, J. Metallization and proximity superconductivity in topological insulator nanowires. *Phys. Rev. B* **105**, 155413 (2022).

[46] Reeg, C., Dmytruk, O., Chevallier, D., Loss, D. & Klinovaja, J. Zero-energy Andreev bound states from quantum dots in proximitized Rashba nanowires. *Phys. Rev. B* **98**, 245407 (2018).

[47] Escribano, S. D., Yeyati, A. L. & Prada, E. Interaction-induced zero-energy pinning and quantum dot formation in Majorana nanowires. *Beilstein J. Nanotechnol.* **9**, 2171–2180 (2018). 30202687 10.3762/bjnano.9.203 PMC6122061

[48] Cayao, J. & Burset, P. Confinement-induced zero-bias peaks in conventional superconductor hybrids. *Phys. Rev. B* **104**, 134507 (2021).

[49] Bordin, A. et al. Tunable crossed Andreev reflection and elastic cotunneling in hybrid nanowires. *Phys. Rev. X* **13**, 031031 (2023). 10.1103/PhysRevLett.132.056602 38364137

[50] Taskin, A. A., Ren, Z., Sasaki, S., Segawa, K. & Ando, Y. Observation of Dirac holes and electrons in a topological insulator. *Phys. Rev. Lett.* **107**, 016801 (2011). 21797561 10.1103/PhysRevLett.107.016801

[51] Huang, Y. & Shklovskii, B. I. Disorder effects in topological insulator nanowires. *Phys. Rev. B* **104**, 054205 (2021).

[52] Schindele, J., Baumgartner, A. & Schönenberger, C. Near-unity Cooper pair splitting efficiency. *Phys. Rev. Lett.* **109**, 157002 (2012). 23102354 10.1103/PhysRevLett.109.157002

[53] Groth, C. W., Wimmer, M., Akhmerov, A. R. & Waintal, X. Kwant: a software package for quantum transport. *New J. Phys.* **16**, 063065 (2014).

[54] Feng, J. et al. Data for “Long-range crossed Andreev reflection in a topological insulator nanowires proximitized by a superconductor". *Zenodo*10.5281/zenodo.12611921 (2024). 10.1038/s41567-025-02806-y PMC12084150 40386803

