# Supplementary material for: Long-range crossed Andreev reflection in a topological insulator nanowire proximitized by a superconductor
Source: Nat Phys. 2025 Mar 11;21(5):708–15. doi: 10.1038/s41567-025-02806-y (PMC12084150; doi:10.1038/s41567-025-02806-y)
Supplement: Supplementary file 1 — Supplementary Figs. 1–7 and Notes 1–8. [file 41567_2025_2806_MOESM1_ESM.pdf]

nature physics

Supplementary information https://doi.org/10.1038/s41567-025-02806-y

Long-range crossed Andreev reflection in a topological insulator nanowire proximitized by a superconductor

In the format provided by the authors and unedited

Supplementary Information:
Long-range crossed Andreev reflection in a topological insulator nanowire proximitized by a superconductor

Contents

**Supplementary Note 1**	**Measurement circuit**	**2**
**Supplementary Note 2**	**Background subtraction in the AB-oscillation data**	**3**
**Supplementary Note 3**	**Temperature dependence of the normal-state oscillations**	**4**
**Supplementary Note 4**	**Conductance matrix in parallel magnetic fields**	**5**
**Supplementary Note 5**	G_{RR} **and** G_{LR} **in the 1.5** μ**m device**	**6**
**Supplementary Note 6**	**Modeling of chemical-potential-dependent local conductance**	**7**
**Supplementary Note 7**	**Zoom-in on the data shown in Fig. 1c of the main text**	**11**
**Supplementary Note 8**	**Asymmetry between** G_{LR} **and** G_{RL} **in Fig. 3 of the main text**	**12**

Supplementary Note 1   Measurement circuit

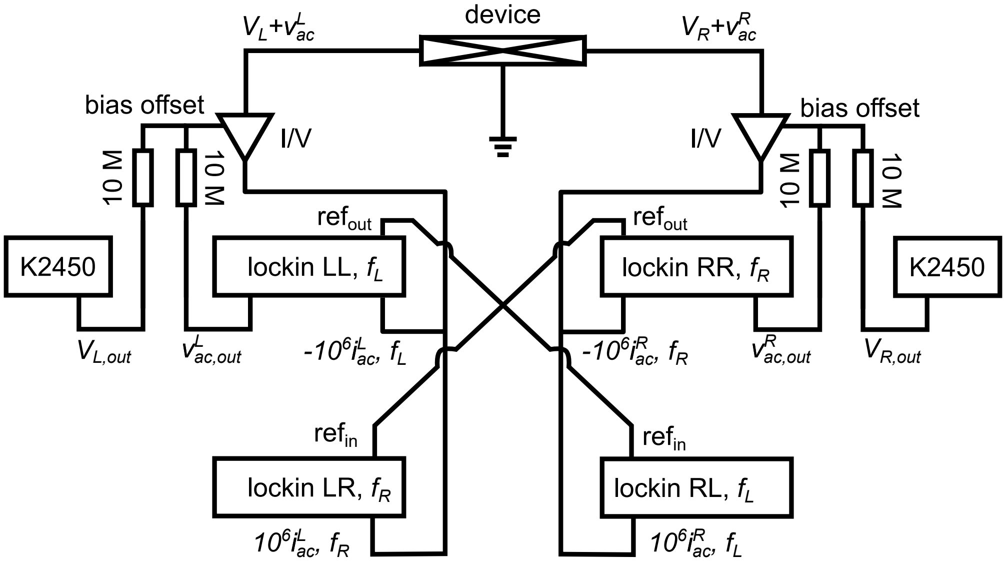

Figure S1: **Sketch of the measurement circuit.** By using different frequencies for the left and the right electrodes, we can measure the conductance matrix in the same circuit[1,2]. We use Keithley DC source K2450 to generate DC voltages and lock-ins (Stanford Research Systems SR860 or NF LI5640) to generate ac voltages. The voltage outputs from these instruments, $V_{\mathrm{L,out}}$ of DC voltage and $v^{\mathrm{L}}_{\mathrm{ac,out}}$ of ac voltage at frequency f_{L}, are applied to 10-MΩ resistors separately, and then supplied to the bias offset port of the amplifier LSK389A from Basel Precision Instruments. The bias offset port is grounded via a 10-kΩ resistor, such that $V_{\mathrm{L,out}}$ and $v^{\mathrm{L}}_{\mathrm{ac,out}}$ are converted to $V_{\mathrm{L}} = V_{\mathrm{L,out}}/1000$ and $v^{\mathrm{L}}_{\mathrm{ac}} = v^{\mathrm{L}}_{\mathrm{ac,out}}/1000$ and the total bias voltage $V_{\mathrm{L}} + v^{\mathrm{L}}_{\mathrm{ac}}$ is applied to the sample through the amplifier, which is connected to the left electrode of the device under test. The local ac current $i^{\mathrm{L}}_{\mathrm{ac}}$ is amplified by the left amplifier and measured by the lock-in named “LL” at the frequency f_{L}. The corresponding nonlocal ac current $i^{\mathrm{R}}_{\mathrm{ac}}$, which is driven by $V_{\mathrm{L}} + v^{\mathrm{L}}_{\mathrm{ac}}$ at the frequency f_{L}, is amplified by the right amplifier and measured by the lock-in named “RL” using the reference signal taken from the lock-in “LL”. The G_{LL} and G_{RL} can be calculated with $G_{\mathrm{LL}} = i^{\mathrm{L}}_{\mathrm{ac}}/v^{\mathrm{L}}_{\mathrm{ac}}$ and $G_{\mathrm{RL}} = i^{\mathrm{R}}_{\mathrm{ac}}/v^{\mathrm{L}}_{\mathrm{ac}}$, both measured at the frequency f_{L}. The G_{RR} and G_{LR} are measured and calculated in a symmetric manner but with a different ac frequency f_{R}.

Supplementary Note 2 Background subtraction in the AB-oscillation data

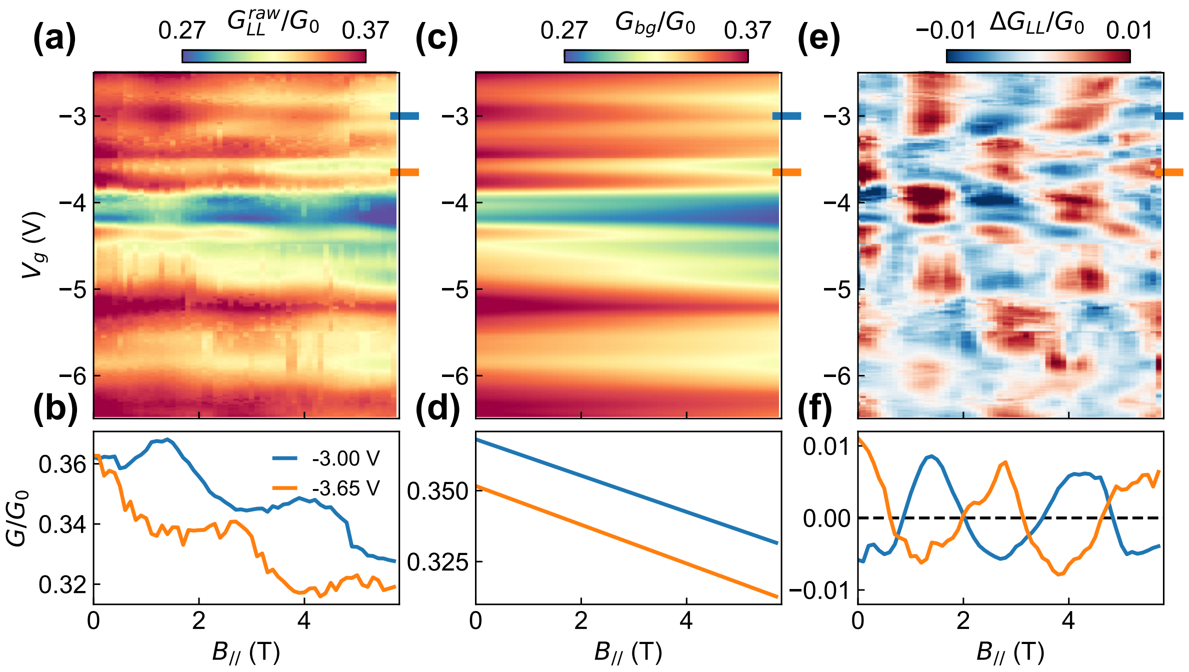

Figure S2: **(a,b)** Raw data of the "checkerboard" pattern shown in Fig. 1b of the main text. The V_g-dependent oscillations are already visible in the raw data. Two representative line-cuts at V_g = -3.00 V and -3.65 V are plotted in (b), where one can see the Aharonov-Bohnm (AB)-like oscillations with phase shifts. **(c,d)** We fitted a linear $B_\parallel$-dependence $G_{\rm bg} = aB_\parallel + b$ for the background $G_{\rm bg}(B_\parallel)$ at each V_g and subtracted it from the raw $G_{\rm LL}^{\rm raw}(B_\parallel)$ data to obtain $\Delta G_{\rm LL} \equiv G_{\rm LL}^{\rm raw} - G_{\rm bg}$ at each V_g. The $G_{\rm bg}(B_\parallel, V_g)$ behavior used for the analysis is shown in (c) and the line-cuts at V_g = -3.00 V and -3.65 V are plotted in (d). **(e,f)** The obtained $\Delta G_{\rm LL}$ and two line-cuts at V_g = -3.00 V and -3.65 V. Noise spikes caused by the gate sweeping have been removed from the $\Delta G_{\rm LL}$ data using the Savitzky–Golay filtering (window length 7 data points, order 3).

Supplementary Note 3   Temperature dependence of the normal-state oscillations

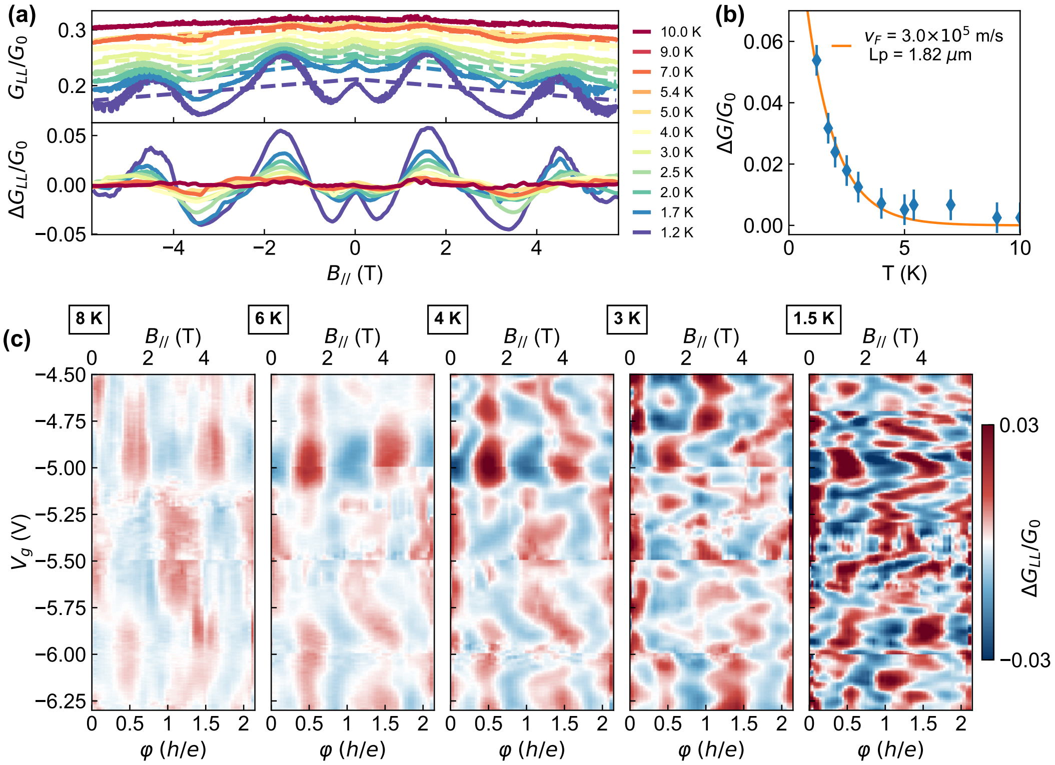

Figure S3: **(a)** AB-like oscillations at $V_\mathrm{g} = -4.37$ V. In the upper panel, the raw data (solid lines) and the linear background (dashed lines) are shown for various temperatures from 1.2 to 10 K. In the lower panel, the ΔG_LL data after the background subtraction and Savitzky-Golay filtering are plotted. **(b)** Oscillation amplitude extracted at $\varphi = -1.6$ T, where the amplitude was maximum. The error bars are due to the error in the magnitude of i_ac caused by the noise in the lock-in measurements, and they are estimated to be the same $0.005G_0$ for all the data points. By using the formula[3] $\Delta G_\mathrm{LL} \propto \exp\left(-k_B T L_\mathrm{p}/\hbar v_\mathrm{F}\right)$, we can extract the phase coherent length $L_\mathrm{p} = 1.82$ μm for $v_\mathrm{F} = 3\times10^5$ m/s. **(c)** Change of the checkerboard pattern upon decreasing the temperature from 8 K to 1.5 K. The checkerboard pattern breaks up into more fine-structured patterns at lower temperature, which is presumably due to the resonance states caused by Fabry-Perot-like interference in the N-section of the TINW, which have the energy-level spacing of about 0.5 meV (see main text).

Supplementary Note 4 Conductance matrix in parallel magnetic fields

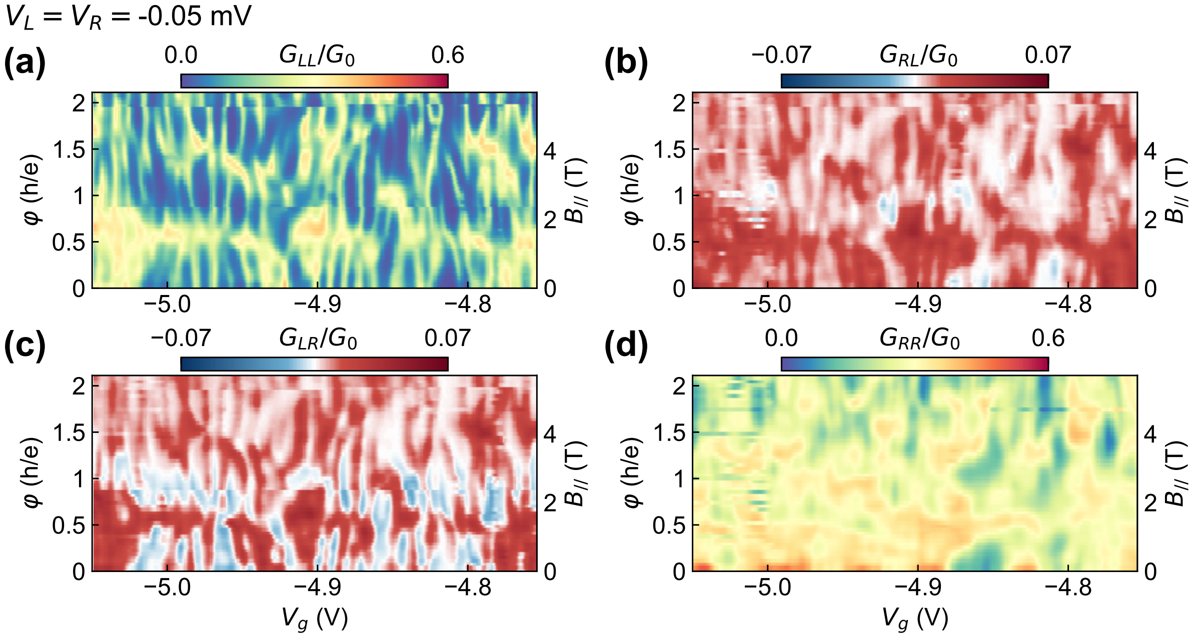

Figure S4: **(a)-(d)** G_{LL}, G_{RL}, G_{LR} and G_{RR} measured at 17 mK with $V_{\mathrm{L}} = V_{\mathrm{R}} = -0.05$ mV as a function of φ (magnetic flux created by $B_{\parallel}$) and V_{g}. The small bias voltage is applied to drive the device into the Cooper-pair splitting regime, such that the CAR process in G_{RL} and G_{LR} is promoted, while keeping G_{LL} and G_{RR} to be similar to their zero-bias values. At around $\varphi = \frac{1}{2}(h/e)$, G_{RL} and G_{LR} are mostly dominated by ECT, but CAR is still visible at some V_{g} values, suggesting that the induced superconductivity in S' does not disappear for $\varphi = \frac{1}{2}(h/e)$.

Supplementary Note 5 G_{RR} **and** G_{LR} **in the 1.5** μ**m device**

In Fig. 6 of the main text, the G_{LL} and G_{RL} data obtained from the 1.5 μm device are shown. To present the complete conductance matrix, Fig. S5 shows the remaining two components, G_{RR} and G_{LR}, measured at the same time with $V_{\mathrm{L}} = V_{\mathrm{R}}$. Negative G_{RL} signifying the CAR process is clearly observed.

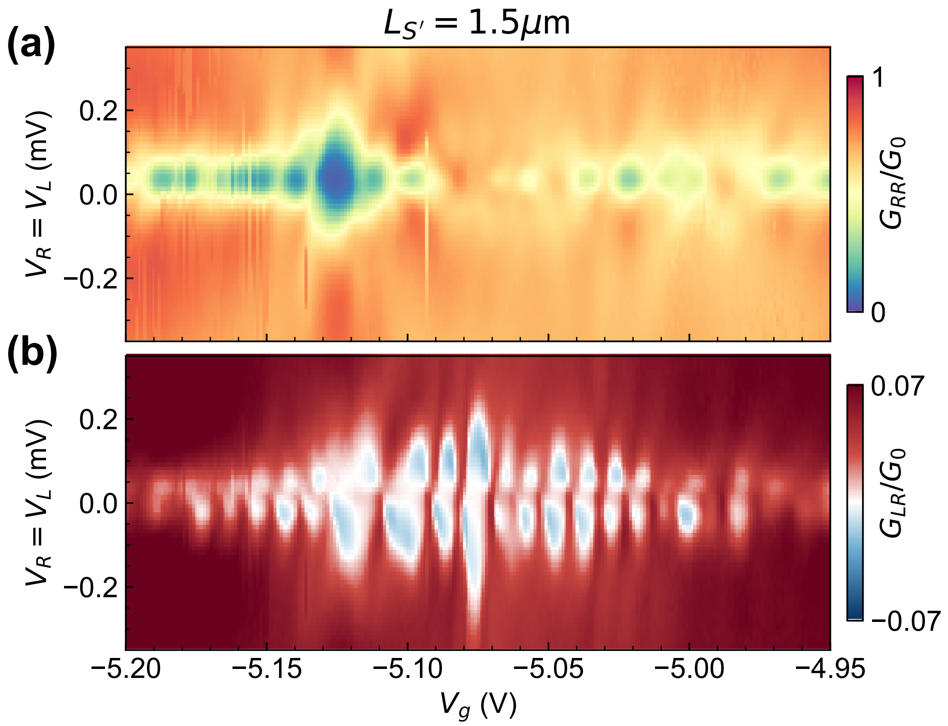

Figure S5: **(a)** G_{RR} and **(b)** G_{LR} of the 1.5 μm device measured at 17 mK in 0 T with $V_{\mathrm{R}} = V_{\mathrm{L}}$.

Supplementary Note 6 Modeling of chemical-potential-dependent local conductance

To model the Fabry-Perot-like oscillations of the Andreev bound state (ABS) energy and corresponding local conductance, G_{LL}, shown in Fig. 2 of the main text, we use the software package KWANT[4]. In particular, we employ a one-dimensional model similar to that discussed in Refs. 5 & 6, such that the Hamiltonian of the one-dimensional nanowire is given by

$$H = \sum_{n=1}^{N} \left[\sum_{\sigma} \left(c^{\dagger}_{n,\sigma} \left\{ t_{n+\frac{1}{2}} + t_{n-\frac{1}{2}} - \mu_n + \gamma_n \right\} c_{n,\sigma} - t_{n+\frac{1}{2}} c^{\dagger}_{n,\sigma} c_{n+1,\sigma} \right) + \Delta_n c^{\dagger}_{n,\downarrow} c^{\dagger}_{n,\uparrow} + \text{ H.c.} \right], \tag{S1}$$

here $c^{\dagger}_{n,\sigma}$ ($c_{n,\sigma}$) creates (annihilates) an electron with spin $\sigma = \uparrow, \downarrow$ at the lattice site n. The total number of sites is given by $N = N_n + 2N_b + N_{\text{SC}}$, where N_n is the number of sites in the normal section on the left of the nanowire, N_b is the number of sites in the tunnel barriers on the left and right of the nanowire that are used to tune the strength of coupling to the leads, and N_{SC} is the number of sites in the superconducting section (see Fig. S6). For our simulations we choose $(N_{\text{SC}}, N_n, N_b) = (400, 98, 9)$ sites. Although our aim is to provide a minimal model of Fabry-Perot-like oscillations and not simulate our precise experimental setup, for our chosen parameters these number of sites will broadly correspond to the lengths of the corresponding sections in our experiment (see below). Note that the quantization of the transverse modes into subbands indexed by angular momentum assures the separability of the transverse physics from longitudinal physics in TINW[7,8], justifying the use of the one-dimensional model to capture the key features in the longitudinal physics.

The parameters t_n and μ_n in Eq. (S1) denote the nearest-neighbour hopping matrix element

and the chemical potential, respectively. The value of these depends on the specific section of the nanowire. In particular, when n corresponds to a normal site or the tunnel barrier the hopping is given by $t_n = t_N$ and in the superconducting section it is given by $t_n = t_S$. Similarly, the chemical potential in the normal section is given by $\mu_n = \mu_N$, in the superconducting section by $\mu_n = \mu_S$, and in the tunnel barriers by $\mu_n = \mu_N + \gamma$, with γ the barrier height. In our experiment there is no well defined tunnel barrier so in our simulations we consider only small barrier heights γ with large broadening by the leads (see below) . The superconducting pairing potential is $\Delta_n = \Delta_0$ in the superconducting section and is zero otherwise. Note, for simplicity we do not include a spin-splitting of the band due to spin-orbit coupling (SOC) since it is unimportant for our discussion of the qualitative features due to Fabry-Perot physics. The inclusion of SOC does not alter the main mechanism of the Fabry-Perot resonance[6], but SOC is important for the realization of the topological phase[5] and its spatial variation is also important for the emergence of the Andreev bands[6] in Rashba nanowires. In TINWs, the way how SOC enters the problem is different but its role in the topological physics is similar[7,8].

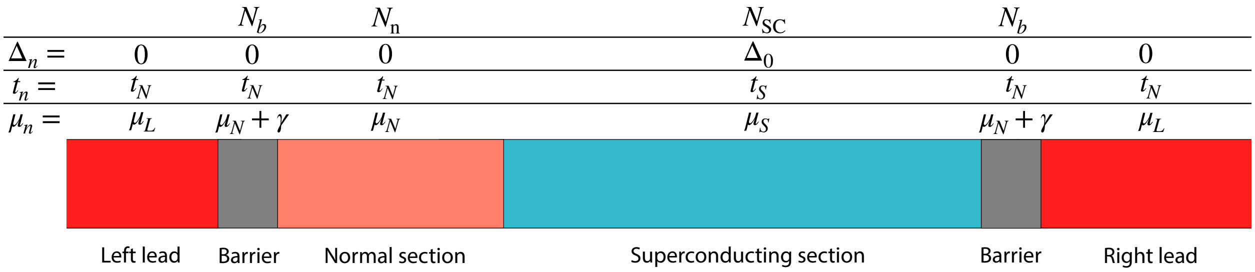

Figure S6: Schematic of one-dimensional nanowire model hosting an Andreev bound state in normal section. The number of sites and the correspondence of parameters t_n, Δ_n, and μ_n in Eq. (S1) are given in the table above the relevant section.

For the simulations in the main text, we choose $(t_N, t_S) = (50, 25)$ meV, $\Delta_0 = 0.4$ meV, $(\mu_N, \mu_S, \gamma) = (2 + \mu, 40, 5)$ meV where μ adjusts the chemical potential in the normal section and barrier (see Fig. 2 of main text). As above, we note that, although our focus in these simulations is on the qualitative features, our chosen values are broadly consistent with the length-scales and energies expected in our experiment. For instance, in our experiment the superconducting section has a length $L_S \sim 1.5$ μm which would correspond to a lattice spacing of $a = L_S/N_{\mathrm{SC}} = 3.5$ nm resulting in a Fermi-velocity in the superconducting section of $v_F = 2\sqrt{t_S\mu_S}a/\hbar \approx 3.5 \times 10^5$ m/s.

To compute conductance normal leads are attached on the left and right ends of the nanowire and are modelled by the same Hamiltonian as the normal sections ($\Delta_n = 0$, $t_n = t_N$) but with fixed chemical potentials μ_L. For our simulations we take $\mu_L = 40$ meV i.e. the same as in the superconducting section. Throughout we focus on the local conductance in the left lead only since this is where we model the normal section at the junction to the lead, an equivalent normal section on the right of the nanowire would yield identical results for G_{RR}. As discussed in the main text, attaching leads broadens the ABS energy which, depending on the strength of the coupling of the ABS to the lead, can result in local conductance at zero-bias, even if the ABS energy does not reach zero energy when no leads are attached. Although the majority of broadening is due to strong coupling to the leads, further a small broadening also occurs due to temperature, which we include via the Fermi-Dirac distribution[6] and take to be $T = 20$ mK.

Finally we note that we are primarily interested in the qualitative physics of Fabry-Perot-like oscillations of ABS energy and, as such, here we utilise a purely one-dimensional model for a

nanowire with a single (spin degenerate) subband that results in a single (spin degenerate) Andreev bound state in the normal section. This is the minimal model required to reproduce oscillations in local conductance as a function of chemical potential that are observed in our experiment. In reality, however, there will be multiple subbands each with different v_F and k_F values. Each subband will contribute to the Andreev bound state spectrum, with all contributions exhibiting Fabry-Perot-like oscillations of energy with various amplitudes, chemical potential dependences, and broadening from the leads. The simulation results are shown in Figs. 2c-2e of the main text.

Supplementary Note 7 Zoom-in on the data shown in Fig. 1c of the main text

Since the 17-mK data shown in Fig. 1c of the main text look like noise, to demonstrate that the strong conductance fluctuations are not due to noise but due to fast V_g-dependent oscillations, a zoom-in on the data is presented in Fig. S7. Note that the measurements for Fig. 1c and Fig. 1d of the main text were performed separately, and the unavoidable gate-sweep hysteresis makes the details of the two data set slightly different.

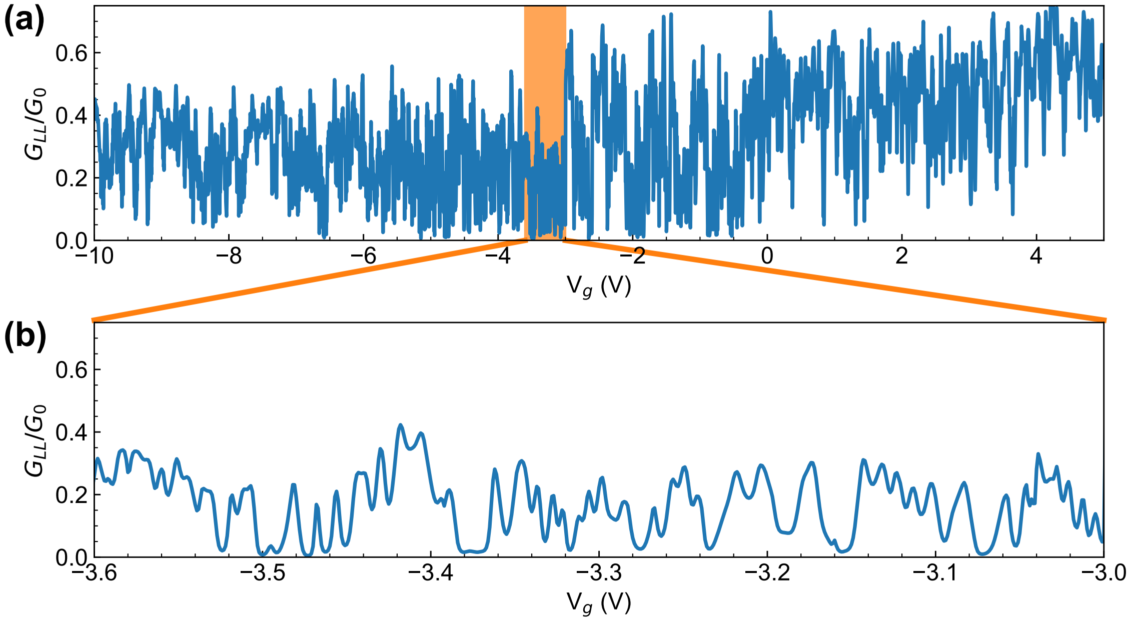

Figure S7: Zoom-in of the G_{LL} vs V_{g} data presented in Fig. 1c of the main text for a narrower V_{g}-range of -3.6 V to -3.0 V.

Supplementary Note 8 Asymmetry between G_{LR} and G_{RL} in Fig. 3 of the main text

It is known that differences in the local conductance can affect the nonlocal conductance, such that G_{LR} and G_{RL} are not the same (but must still respect certain relations from symmetries). Detailed discussions on the conductance symmetries of multiterminal devices can be found in Refs. 9, 10. In the present case, we found experimentally that G_{RL} and G_{LR} depend strongly on the local conductance where the current is measured. For example, for G_{LR} shown in Fig. 3f of the main text, the current was measured from the left, where G_{LL} was off-resonance and the ECT rate was low, making CAR to be easily visible. On the other hand, for G_{RL} shown in Fig. 3e, the current was measured from the right, where G_{RR} was not exactly at off-resonance and the ECT rate was higher; this caused a positive offset in G_{RL}, smearing the CAR signal.

References

1. Gramich, J., Baumgartner, A. & Schönenberger, C. Andreev bound states probed in three-terminal quantum dots. *Phys. Rev. B* **96**, 195418 (2017).

2. Ménard, G. C. *et al.* Conductance-matrix symmetries of a three-terminal hybrid device. *Phys. Rev. Lett.* **124**, 036802 (2020).

3. Dufouleur, J. *et al.* Quasiballistic transport of Dirac fermions in a $\mathrm{Bi_2Se_3}$ nanowire. *Phys. Rev. Lett.* **110**, 186806 (2013).

4. Groth, C. W., Wimmer, M., Akhmerov, A. R. & Waintal, X. Kwant: a software package for quantum transport. *New J. Phys.* **16**, 063065 (2014).

5. Reeg, C., Dmytruk, O., Chevallier, D., Loss, D. & Klinovaja, J. Zero-energy Andreev bound states from quantum dots in proximitized Rashba nanowires. *Phys. Rev. B* **98**, 245407 (2018).

6. Hess, R., Legg, H. F., Loss, D. & Klinovaja, J. Local and nonlocal quantum transport due to Andreev bound states in finite Rashba nanowires with superconducting and normal sections. *Phys. Rev. B* **104**, 075405 (2021).

7. Legg, H. F., Loss, D. & Klinovaja, J. Majorana bound states in topological insulators without a vortex. *Phys. Rev. B* **104**, 165405 (2021).

8. Legg, H. F., Loss, D. & Klinovaja, J. Metallization and proximity superconductivity in topological insulator nanowires. *Phys. Rev. B* **105**, 155413 (2022).

9. Danon, J. *et al.* Nonlocal conductance spectroscopy of Andreev bound states: Symmetry relations and BCS charges. *Phys. Rev. Lett.* **124**, 036801 (2020).

10. Maiani, A., Geier, M. & Flensberg, K. Conductance matrix symmetries of multiterminal semiconductor-superconductor devices. *Phys. Rev. B* **106**, 104516 (2022).
